# Polymorphism rs1108580 in DBH gene is associated with the risk of depression in alcohol-dependent patients

**DOI:** 10.1192/j.eurpsy.2022.735

**Published:** 2022-09-01

**Authors:** A. Nikolishin, N. Chuprova, A. Kibitov

**Affiliations:** Serbsky National Medical Research Center on Psychiatry and Addictions, Molecular Genetics Laboratory, Moscow, Russian Federation

**Keywords:** Alcohol dependence, Dopamine, Genetics, Depression

## Abstract

**Introduction:**

Alcohol dependence and depression are often combined, patients with comorbid pathology have a more severe course of the disease, a high risk of suicide and therapeutic resistance. Enzyme dopamine-beta-hydroxylase (DBH) is a key player in a link between dopamine and norepinephrine neuromediations and may be involved in alcohol dependence and depression comorbidity and genetic markers in DBH gene may be associated with the risk of comorbid state.

**Objectives:**

To test an association of DBH gene polymorphisms rs161111580 and rs1108580 with depression risk in alcohol-dependent patients.

**Methods:**

Our sample consisted of 104 inpatients diagnosed by ICD-10 criteria: 40 with alcohol dependence (AD group) (age 45.6 (SD 10.853), 5% females), 64 with depression and alcoholism comorbidity (AD+D group) (age 41.2 (SD 9.903), 22% females) and 112 healthy controls (age 35.5 (SD 8.286), 15% females). rs1108580 and rs1611115 were detected by RT-PCR.

**Results:**

For rs161111580, frequencies of minor T allele (p=0.031) and ТТ genotype (p=0.017) was higher, СС genotype (p=0.042) was lower in AD group vs. controls. rs161111580 T allele and TT genotype increases the risk of AD (OR=3.715, 95%CI [1.728-7.986], P=0.001 and OR=4.009, 95%CI [1.502-10.699], P=0.006). For rs161111580, frequency of ТТ genotype (p=0.009) was higher in AD+D group vs. controls. For rs1108580, frequency of major А allele (p=0.059, trend) was higher in AD+D, then in AD group. Major А allele rs1108580 increases the risk of depression in alcohol-dependent patients (OR=2.74, 95%CI [1.283-5.855], P=0.001).

**Conclusions:**

It was shown that the DBH rs1108580 increases the risk of depression in patients with alcohol dependence.

**Disclosure:**

No significant relationships.

